# Analysis of the Efficacy of Two Treatment Protocols for Patients with Symptomatic Oral Lichen Planus: A Randomized Clinical Trial

**DOI:** 10.3390/ijerph18010056

**Published:** 2020-12-23

**Authors:** Simona Santonocito, Alessandro Polizzi, Rocco De Pasquale, Vincenzo Ronsivalle, Antonino Lo Giudice, Gaetano Isola

**Affiliations:** 1 Department of General Surgery and Surgical-Medical Specialties, Unit of Oral Pathology, School of Dentistry, University of Catania, 95124 Catania, Italy; simonasantonocito93@gmail.com (S.S.); alexpoli345@gmail.com (A.P.); vincenzo.ronsivalle@hotmail.it (V.R.); nino.logiudice@gmail.com (A.L.G.); 2 Department of General Surgery and Surgical-Medical Specialties, Unit of Dermatology, University of Catania, 95124 Catania, Italy; r.depasquale@unict.it

**Keywords:** oral lichen planus, clobetasol, oral solution, calcium hydroxide, hyaluronic acid, umbelliferone, oligomeric pro-anthocyanidins, therapy, Thongprasom’s Score

## Abstract

Oral lichen planus (OLP) is a chronic, inflammatory, immune-mediated disease, which can alter the quality of life of patients. The aim of this randomized clinical trial was to compare the therapeutic efficacy of clobetasol oral gel 0.05% versus an anti-inflammatory in oral solution (mouthwash) in the management of patients suffering from symptomatic OLP. The secondary objective was to analyze which one of the two treatments induced a greater risk of developing side effects. Forty patients were assigned (20 patients for group), through a randomized design, to receive clobetasol gel 0.05% or an anti-inflammatory mouthwash, which contains calcium hydroxide, hyaluronic acid, umbelliferone and oligomeric pro-anthocyanidins) for three months. At baseline (T0) and after 3 months (T1), patients underwent dental and dermatological examinations to assess their symptoms (Numerical Pain Scale (NRS) score) and signs (Thongprasom score). Data were calculated using *T*-test for the dependent variable, Wilcoxon test and Mann-Whitney u test. Both clobetasol and anti-inflammatory resulted in a statistically significant reduction of signs, (*p* < 0.001 and *p* = 0.02, respectively) and symptoms (*p* < 0.001 for clobetasol and *p* = 0.02 for anti-inflammatory). In conclusion, the results evidenced that, compared to clobetasol, the anti-inflammatory was less effective in determining the reduction of signs and symptom in OLP patients.

## 1. Introduction

Oral lichen planus (OLP) is a chronic inflammatory disease, which affects the stratified scaly epithelium of the oral mucosa and the underlying lamina propria. It may be accompanied by skin manifestations and lesions of the genital mucosa [1]. It is one of the most common dermatological pathologies that afflict the oral cavity with an estimated prevalence between 0.22% and 5% of the world’s population and an incidence of 2.2% with a ratio of male to female of 2:1. The age of onset is between 30 and 70 years of age, with very rare clinical cases in very young and pediatric ages [2,3]. The etiology and pathogenetic mechanisms remain unknown [2]. Recent evidence supports a central role of immune dysregulation in the pathogenesis of OLP, reflected by an altered production of inflammatory mediators both locally and systemically [4].

The lesions have distinctive clinical features and a characteristic bilateral distribution [5,6]. The most commonly affected area is the buccal mucosa, followed by tongue and gum [7]. The symptoms range from mild discomfort to intense burning and pain. Atrophic/erythematous and erosive forms are most commonly the cause of pain and soreness, which interfere with chewing, phonation and swallowing, also leading to severe functional limitations [8].

One of the most important problems in the management of OLP is its chronic-recurrent nature, which requires long-term therapy. It is currently impossible to achieve complete eradication of the disease with the available methods [9], as there is little data on the long-term therapeutic results of OLP patients and there is no definitive treatment that would result in long-term remission [10]. The treatment must be aimed at achieving specific objectives, such as the elimination/reduction of atrophic and ulcerative lesions, the alleviation of symptoms and the potential reduction of the risk of malignant transformation. The degree of clinical involvement, the type of predominant clinical lesions, the patient’s symptoms and age and possible previous therapeutic failures must be considered when planning the pharmacological treatment. Almost all published reviews agree that only erosive/ulcerative or symptomatic forms should be treated. Asymptomatic reticular lesions, on the other hand, generally do not require therapy but must be subject to constant follow-up [11,12]. It is also recommended to eliminate any irritants or aggravating factors in the oral cavity, such as occlusion problems, poor oral hygiene, and to avoid smoking, alcohol, irritating food and drink [13]. The drugs used to treat OLP are glucocorticoids, immunosuppressants (cyclosporine) [5,14], tacrolimus [5,14,15], pimecrolimus [16,17]) and immunomodulators (retinoic acid) [18,19,20], and few have been developed directly for oral use [21]. High potency topical steroids are currently used in first-line therapy, as they have fewer side effects than systemic agents [22]. Systemic agents are required when there are lesions in extraoral sites, or OLP forms resistant to topical treatments [23]. Clobetasol propionate appears to be the most effective topical steroid, as 56–75% of patients treated with it on an adhesive basis have undergone complete remission [24,25], while this percentage drops to 30–15% for other corticosteroids [10].

Long-term use of high potency topical steroids may lead to the development of collateral effects, including candidiasis, burning sensation, mucosal atrophy, bad taste, nausea, sore throat and dry or swollen mouth [26,27,28,29]. Cases of systemic absorption and adrenal suppression following high potency topical and systemic corticosteroid therapy have been reported, especially when used in the long-term management of chronic diseases such as OLP [30]. It was precisely the need to find safer and more effective drugs for the treatment of symptomatic OLP that motivated research to evaluate possible therapeutic alternatives aloe vera, curcuminoids, hyaluronic acid, lycopene, psychiatric therapy, topical thalidomide and low-intensity laser therapy [31]. An anti-inflammatory in oral solution, in the form of mouthwash, containing hyaluronic acid and other active ingredients with anti-inflammatory and antibacterial activity, such as calcium hydroxide, oligomeric pro-anthocyanidins and umbelliferone, is currently used in the treatment of various oral mucosal disorders, such as gingivitis, periodontitis, recurrent aphthosis, urethral mouth syndrome, radiotherapy stomatitis and chemotherapy. Hyaluronic acid has aroused considerable interest, as several studies have indicated that it has significant benefits in the management of OLP [32,33,34]. The antibacterial activity of calcium hydroxide and umbelliferone can be used in the treatment of Lichen as alterations in the oral microbiota are among its potential etiological factors [35].

The primary objective of this study was to compare the therapeutic efficacy of clobetasol propionate 0.05% oral gel versus an anti-inflammatory mouthwash in an oral solution for the management of patients suffering from symptomatic OLP. The secondary objective was to analyze which one of the two treatments induced a greater risk of developing side effects. The null hypothesis is that there is no difference in efficacy between the two protocols in determining an improvement in OLP or in the development of side effects.

## 2. Materials and Methods

### 2.1. Study Design

**Figure 1:**
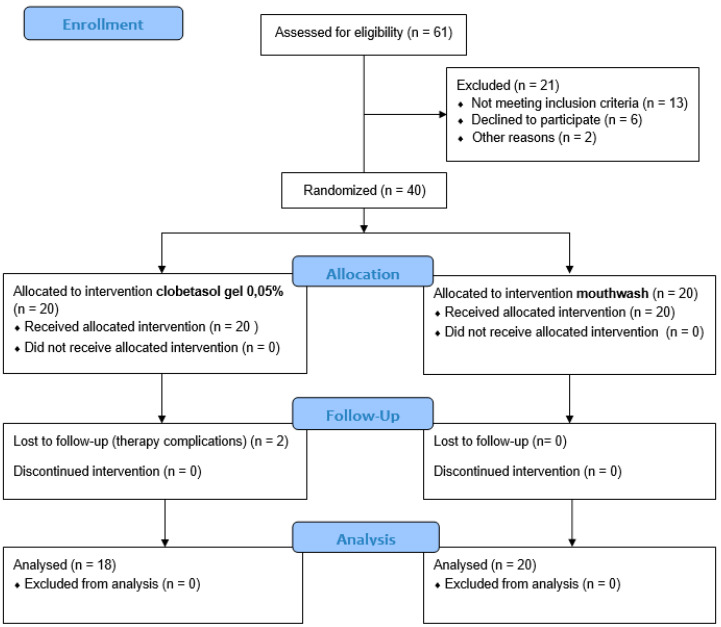
Flow chart of the study.

The study was designed as a randomized controlled clinical trial (RCT). The patients included in the study were enrolled at the School of Dentistry of the Department of General Surgery and Surgical-Medical Specialties, University of Catania, Catania, Italy, between June 2019 and February 2020. The local International Review Board (IRB) of the University of Catania approved the study protocol (prot. 121/120/PO). The study was registered on clinicaltrials.gov (NCT04673916). Before the study, all patients signed written informed consent. The study was performed following the guidelines of the Declaration of the World Medical Association 1975 in Helsinki, revised in 2000. This trial was conducted in agreement with the CONSORT guidelines (Figure 1).

The inclusion criteria were: (1) age ≥ 18 years; (2) clinical diagnosis and histological diagnosis of OLP on the basis of WHO criteria; (3) presence of symptoms related to OLP. Exclusion criteria were: (1) presence of systemic conditions that may have affected the study results; (2) state of pregnancy or breastfeeding; (3) histological signs of dysplasia; (4) drugs inducing a lichen response (Angiotensin-converting-enzyme (ACE)-inhibitors, β-blockers, etc.); (5) treatment of OLP in the six months prior to the start of the programme; (6) presence of extraoral lichenoid lesions (genital, cutaneous and other); (7) history of previous immunodeficiency or HIV seropositivity; (8) previous allogeneic bone marrow transplantation; (9) presence of systemic lupus erythematosus or other autoimmune diseases; (10) current orthodontic therapy; (11) use of incongruous removable dentures.

At baseline, a total of 61 patients were enrolled. However, after the first screening, 21 patients were excluded because they did not meet the inclusion criteria (*n* = 21), declined to participate (*n* = 6) or were absent at the first visit (*n* = 2). Finally, a total number of 40 patients (14 female and 12 male) with symptomatic OLP, aged between 27 and 80 years (mean age of 71.91), were enrolled and assigned to the clobetasol group (*n* = 20) and the anti-inflammatory group (*n* = 20).

### 2.2. Power Sample Size and Randomization

At baseline, a power sample analysis was performed. The calculation of the sample size was established, having at least 32 individuals (18 for the arm), for an alpha error of 0.05 and a power of 80%. Considering potential drop-out (e.g., patients lost during the follow-up sessions), 20 patients per group were finally enrolled.

After the baseline examination, the enrolled subjects were randomly assigned to one of the two treatment protocols using a computer-generated table. Allocation concealment was ensured by a clinician not involved in the subsequent study phases and by providing sealed envelopes (containing assignments for individual patients) to the clinicians who prescribed the treatment. Investigators were blinded to the group assignment.

### 2.3. Treatment Protocols

In all patients, at baseline (T0), subjects were given anamnestic questionnaires before the start of treatment and were instructed and made aware of the dosage and application methods of the two treatments under examination. The clobetasol group was treated with clobetasol propionate 0.05%, while the anti-inflammatory group was treated with mouthwash. The drug used consisted of clobetasol propionate 0.05%, ethyl alcohol 96° (50%), hydroxy-ethyl-cellulose (4%); and preserved water (just enough to 100%) that was topically applied [10]. This drug was produced as a galenic formulation. Clobetasol propionate was applied twice a day (every 12 h) to the lesions with a soft bristle brush. All subjects were advised not to drink or eat during the hour following application of the medication. In patients of the anti-inflammatory group, the mouthwash was used pure and without dilution at a dosage of 20 mL, 3 times a day, immediately after normal daily oral hygiene was prescribed. It contained calcium hydroxide, hyaluronic acid, umbelliferone and oligomeric pro-anthocyanidins. Patients were instructed to rinse for at least five min over the entire oral mucosa, with particular emphasis on the regions where the lesions were located.

Each patient was also reminded to avoid taking food or drink for at least 20 min after using the medicine. All the patients who took part in the study were instructed to avoid cigarette smoking, the consumption of alcoholic beverages, acidic and irritating foods and always to maintain correct oral hygiene. The treatment would be discontinued at any time by the research group if undesirable effects occurred or when patients indicated that the study should be discontinued.

### 2.4. Data Collection

After baseline, patients were followed for three months of therapy. Each patient underwent at both time T0 (baseline) and T1 (after 3 months), a general objective oral examination and an interview using specific medical questionnaires.

The inspection of the oral cavity allowed us to assess the clinical grading of the lesions, by direct measurement, using the scale used by Thongprasom et al. as reference. This gives a score that varies from 0 to 5, using a millimeter reference: 0, in the absence of lesions; 1, in the presence of hyperkeratosis streaks; 2, in the presence of an atrophic area less than 1 mm^2^; 3, in the presence of an atrophic area greater than 1 mm^2^; 4, in the presence of an erosive area less than 1 mm^2^; 5, in the presence of an erosive area greater than 1 mm^2^ [36]. In the presence of multiple injuries, the value was calculated by summing the values of each injury.

A medical questionnaire was compiled for each patient, in which the intensity of the symptoms reported by the patient, in terms of pain and burning, were assessed using the “Numerical Pain Scale” (NRS), in which pain scores ranged from zero (no pain) to 10 (severe pain), with intermediate ranges, 1–3 (mild pain), 4–6 (moderate pain) and 7–9 (severe pain) [37]. The remission of symptoms and signs were evaluated through downstaging of symptoms and signs, respectively. The downstaging of symptoms is the difference between the NRS score at time T0 and T1. The downstaging of signs was given by the difference between the Thongprasom score at time T0 and T1.

### 2.5. Statistical Analysis

The data were first examined for normality by the Kolmogorov-Smirnov test, and subsequently a non-parametric method was performed. The Wilcoxon or t-coupled test was used to detect statistically significant clinical differences within the clobetasol group and the anti-inflammatory group over time. The differences between the groups were tested using the Mann-Whitney U test for independent non-parametric quantitative variables. Statistical analysis was performed using the SPSS Windows package (version 25; SPSS, Chicago, IL, USA).

## 3. Results

The demographic and clinical features are shown in Table 1. Of the 40 patients initially enrolled in the study, two (one patient in the clobetasol group) did not complete the study due to the onset of side effects of the drug used. Therefore, the final group included 38 patients that completed the study. There were no significant differences between the two groups with regard to age and gender.

### 3.1. Primary Endpoint

**Figure 2:**
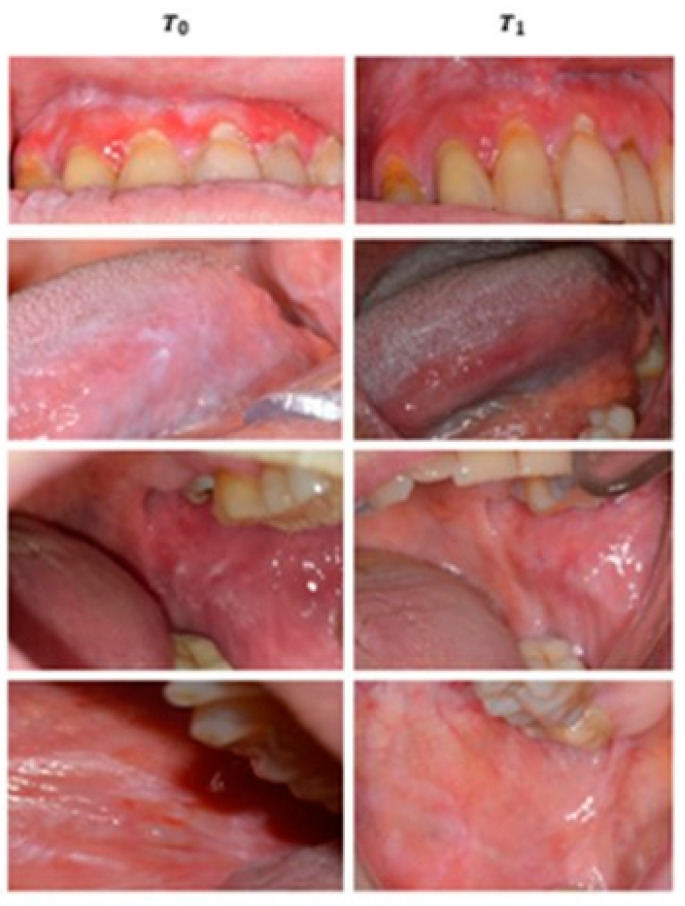
Intra-oral photos of some of the subjects who joined the clinical trial at T0 and T1. The action of the drug on the lesions can be seen after 12 weeks of treatment (Clobetasol group).

**Figure 3:**
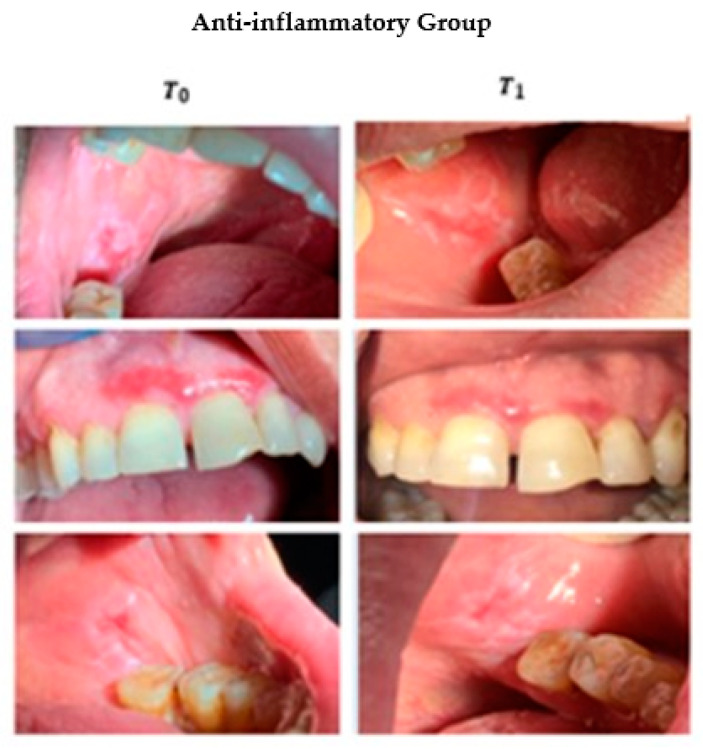
Intra-oral photos of some of the subjects who joined the clinical trial at T0 and T1. The action of the drug on the lesions can be seen after 12 weeks of treatment (Anti-inflammatory group).

Both clobetasol and anti-inflammatory resulted in a statistically significant reduction of signs in the treated groups, with a *p*-value of <0.001 (Wilcoxon test) and 0.02 (Wilcoxon test) respectively (Table 2). More specifically, 16 patients treated with clobetasol (89%) improved after three months of treatment, 13 of whom achieved complete remission of OLP signs (Thongprasom Score < 2) and three partial remission (Thongprasom Score ≥ 2). In two patients (11%) there was an absent remission of OLP signs (Figure 2). Instead of patients treated with anti-inflammatory drugs (mouthwash), 12 showed a reduction in signs (60%) after three months, of which six achieved complete remission (Score Thongprasom < 2) and six achieved partial remission (Score Thongprasom < 2). Eight patients (40%) showed no differences in OLP signs from T0 to T1 (no remission of signs) (Figure 3).

Regarding OLP symptoms, both treatments resulted in a statistically significant reduction in symptoms, with a *p*-value < 0.001 for clobetasol (*T*-test for dependent variables) and 0.02 for anti-inflammatory (Wilcoxon test) (Table 3). More specifically, 16 of 18 patients treated with clobetasol (89%) achieved remission of symptoms, of which only two reported no symptoms (NRS = 0), 12 a mild symptomatology (NRS points between 1 and 3) and four a moderate symptomatology (NRS points between 4 and 5). Only two patients reported no improvement in symptomatology. In the anti-inflammatory group, 17 out of 20 patients treated (85%) showed a reduction in symptoms, of which one patient reported a total absence of symptoms (NRS = 0); 12 patients reported mild symptoms (an NRS score between 1 to 3) and three patients moderate symptoms (an NRS points between 4 to 6). Only three patients (15%) found no remission of symptoms.

The comparison of the downstaging of the OLP signs (the difference between Thongprasom’s score at T0 and T1) between the clobetasol and anti-inflammatory group was carried out with Mann Whitney U test, which indicated that the reduction of signs detected in the two groups is statistically significant, with a *p*-value of 0.009. Moreover, the downstaging of symptoms (difference between the NRS score referred + to T0 and T1) between the clobetasol and anti-inflammatory group was statistically significant (*p* = 0.001) (Table 4).

### 3.2. Secondary Endpoint

Of the 40 patients enrolled in the study, two patients in the clobetasol group did not complete the study due to side effects. Of the 38 patients who completed the trial, four of the 18 patients in the clobetasol group (22.22%) experienced mild side effects, but in no case did drug suspension become necessary. No undesirable effects were reported in the anti-inflammatory group.

## 4. Discussion

This is the first study in which the activity of an anti-inflammatory in oral solution was compared with the activity of clobetasol gel, conventionally used as a first-line drug in the treatment of symptomatic OLP. Active ingredients making up the anti-inflammatory in oral solution have been examined individually in the treatment of OLP: aloe vera, and hyaluronic acid.

Recent OLP therapy studies suggest that high potency topical corticosteroids are the first-line treatment for this disease and indicate clobetasol propionate as the most effective topical steroid [38,39]. However, it should be considered that high-potency topical corticosteroids, when used for long periods or in excessive amounts, can lead to atrophic effects, as they inhibit collagen synthesis in connective tissue [27], and oral candidiasis [26,27]. Current data suggest that adrenal suppression is not a significant side effect in the long-term management of OLP with topical clobetasol propionate (0.5 mg d^−1^) [28,29,40].

Clobetasol is a glucocorticoid with high anti-inflammatory, antiproliferative and immunosuppressive activity with modest mineral-corticoid activity, which allows good management of the disease without exposing the patient to systemic side effects. In this regard, correct treatment can produce a high level of well-being with a minimum incidence of side effects such as lunar face and hirsutism, occasionally reported [41]. Moreover, after six months of follow-up, 65% of patients treated with clobetasol maintained the improvement. Most studies have shown that topical corticosteroids are safe when applied to the mucous membranes for short intervals of time and up to a maximum of six months [24]. Prolonged contact with the oral mucosa should be avoided, as it can damage mucosal barriers and induce local immunosuppression, predisposing to oropharyngeal candidiasis, one of the most common side effects of topical corticosteroid therapy [42]. The literature reports several cases of non-response to treatment, associated with a clinical and symptomatological worsening of the disease [35].

Topical anti-inflammatory in oral solution is currently indicated in the treatment of various oral disorders, such as gingivitis, periodontitis, recurrent aphthosis, burning mouth syndrome, radiotherapy stomatitis and chemotherapy. It contains various active ingredients: calcium hydroxide (lime water 10%); hyaluronic acid; umbelliferon and oligomeric pro-anthocyanidins, obtained from Pinus Pinaster. Calcium hydroxide is a strong base with a powerful antibacterial action: when it comes into contact with saliva, partly made up of water, it releases hydroxide ions, acidifying the environment and causing an increase in pH. This determines the denaturation of proteins and phospholipids in the cell membrane of Gram + bacteria and inhibits the toxic action of some Gram- because it hydrolyzes lipid A, a fundamental constituent element of endotoxins; it has also been seen to act on the biofilm bacterium deposited on the teeth. Its antibacterial action can be used in the treatment of Lichen since alterations in the oral microbiota are among its potential etiological factors [43]. The hyaluronic acid contained in the drug, on the other hand, has a trophic and anti-inflammatory action [44] on the oral mucosa as it has been shown to favor the proliferation of fibroblasts [45], collagen synthesis and the expression of TGF-β, a factor which is able to repress the autoimmune response against self-antigens. Umbelliferon is also a substance with antibacterial activity capable of inhibiting the formation of bacterial biofilm [46] on surfaces. On the other hand, oligomeric pro-anthocyanidins, obtained from Pinus Pinaster, have an anti-inflammatory and antioxidant action [47] because they inhibit cyclooxygenases and phospholipases 2 and their therapeutic use is particularly indicated in the treatment of oral cavity disorders [24]. The drug does not contain chlorhexidine and alcohol but does contain fluorine.

The results of the present study evidenced that both clobetasol and the anti-inflammatory mouthwash induced a statistically significant clinical improvement in the OLP. However, clobetasol appears to be more effective in determining the reduction of clinical signs, as 89% of patients had a reduced Thongprasom score compared to 60% of patients treated with the anti-inflammatory. In addition, 72.2% of clobetasol patients had a complete remission of signs compared to 30% of patients treated with anti-inflammatory drugs. The reduction in symptoms is also statistically significant for both treatments. There are no significant differences in the ability to reduce symptoms between the clobetasol group and the anti-inflammatory group; the reduction in symptoms recorded is 85% and 88%, respectively. Although 35% of patients treated with the anti-inflammatory group did not experience significant clinical improvement, more than half said they felt better, even in the presence of erosions. This agrees with recent literature reports that the quality of life of patients with atrophic/erosive OLP can improve significantly, even in the absence of complete resolution of all oral signs [48,49,50,51,52,53,54]. Clobetasol has been shown to lead to an increased onset of side effects [54,55,56,57]. In 4 of the 18 patients in the clobetasol group (22.22%) minor side effects occurred, but in no case did the discontinuation of the drug become necessary, as these were minor gastrointestinal symptoms. In these cases, greater attention was paid during the application of the drug to avoid ingestion. Two other patients did not have to interrupt the clinical trial. In one female patient, a phenomenon of hypersensitivity to the active ingredient of the drug used was reported, which led to a worsening of symptoms, associated with an increase in erosive areas and spontaneous bleeding; after the interruption of treatment, the clinical lesion returned. Another patient, also female, developed a fungal superinfection, which led to the suspension of treatment for about a fortnight and oral suspension therapy based on Nystatin three times a day; after the subsequent therapy, the patient has been reassessed by the oral dermatologist. In conclusion, anti-inflammatory has proven to be less effective and powerful in determining the reduction of signs in OLP patients. On the other hand, it has not shown substantial differences compared to clobetasol in its ability to induce symptom reduction. The use of the anti-inflammatory has not led to any side effects, unlike clobetasol.

However, the present study has some limitations that need to be addressed. One of these limitations includes the small sample size and the short periods of observation of the sample over time, as it would have been desirable to be able to follow the patients constantly in the post-treatment phase, for at least three months follow-up. This could be useful in understanding how long the beneficial effects induced by the two drugs in question last after the end of treatment. In the literature, it is reported that the clinical improvement reported in patients following treatment with Clobetasol propionate persists over the following six months [41], whereas no information is available on the behaviour of the lesions after suspension of the topical anti-inflammatory drug in oral solution.

## 5. Conclusions

The results of the present study evidenced that:

1. The anti-inflammatory (mouthwash) could be used in the treatment of symptomatic forms of OLP with a Thongprasom score < 2, as it resulted in good symptom control and significant activity in preventing lesion progression.
2. Clobetasol seems to be confirmed once again as the treatment of first choice in the most severe forms of OLP (Thongprasom score > 2), as the study showed that the anti-inflammatory has a limited ability to induce remission of signs in subjects with severe forms of OLP, compared with clobetasol.

## Figures and Tables

**Table 1:** Variation of the oral lichen planus (OLP) sign score after the administration of the two protocols adopted. SD, standard deviation.

Parameter	Clobetasol (*n* = 18)	Anti-Inflammatory Mouthwash (*n* = 20)
Age (years), mean ± SD	65.55 ± 9.61	62.5 ± 9.13
Age (years), range	48–80	32–79
Gender (male/female)	8 a 10	10 a 10
Females/Total (%)	44%	50%
Males/Total (%)	46%	50%
Age males, mean ± SD	63.08 ± 11.47	62 ± 13.67
Age females, mean ± SD	67.88 ± 6.92	62.4 ± 13.07

**Table 2:** Variation of the OLP sign score after the administration of the two protocols adopted.

Scheme					
Treatment	Baseline (T0)		After 3 Months (T1)		*p*-Value
	Median	Min–Max	Median	Min–Max	
Clobetasol	3	1–5	2.5	0–3	<0.001
Anti-inflammatory	1	1–4	1.5	1–3	0.02

**Table 3:** Variation of the OLP symptoms score after the administration of the two protocols adopted. SD, standard deviation.

Symptoms Score (Numerical Pain Score (NRS) Score)			
Treatment	Baseline (T0)	After 3 Months (T1)	*p*-Value
	Mean ± SD	Mean ± SD	
Clobetasol	4.67 ± 2.25	2.33 ± 1.64	<0.001 *
Anti-inflammatory	3.05 ± 1.23	1.85 ± 1.23	0.02 **

* *T*-test for dependent variables; ** Wilcoxon test.

**Table 4:** Comparison of downstaging of symptoms and signs between the clobetasol group and the anti-inflammatory group.

Downstaging Score					
Parameters	Clobetasol		Anti-Inflammatory		*p*-Value
	Median	Min–Max	Median	Min–Max	
Symptoms	3	0–4	1	0–2	0.009 *
Signs	1	0–3	1	0–3	0.001 *

Min–Max: minimum–maximum; * Mann-Whitney U test.

## Data Availability

The study was registered on clinicaltrials.gov (NCT04673916).
